# Genome-wide selective signatures mining the candidate genes for egg laying in goose

**DOI:** 10.1186/s12864-023-09852-x

**Published:** 2023-12-06

**Authors:** Hongchang Zhao, Guobo Sun, Xiaohui Mu, Xiaoming Li, Jun Wang, Mengli Zhao, Gansheng Zhang, Rongchao Ji, Chao Chen, Guangliang Gao, Jian Wang

**Affiliations:** 1 Jiangsu Agri-animal Husbandry Vocational College, Taizhou, 225300 China; 2 National Waterfowl of gene pool, Taizhou, 225511 China; 3 Taizhou Fengda Agriculture and Animal Husbandry Technology Co., Ltd, Taizhou, 225511 China; 4 Chongqing Academy of Animal Science, Chongqing, 402460 China

**Keywords:** Goose, Genetic background, Egg-laying trait, Genetic signatures

## Abstract

**Background:**

Improving the egg production of goose is a crucial goal of breeding, because genetics is the key factor affecting egg production. Thus, we sequenced the genomes of 55 Chinese indigenous geese from six breeds, which were divided into the high egg-laying group (ZE, HY, and SC) and low egg-laying group (ZD, LH, and ST). Based on the results of the inter-population selection signal analysis, we mined the selected genome regions in the high egg-laying germplasm population to identify the key candidate genes affecting the egg-laying traits.

**Results:**

According to the whole-genome sequencing data, the average sequencing depth reached 11.75X. The genetic relationships among those six goose breeds coincided with the breed’s geographical location. The six selective signal detection results revealed that the most selected regions were located on Chr2 and Chr12. In total, 12,051 single-nucleotide polymorphism (SNP) sites were selected in all six methods. Using the enrichment results of candidate genes, we detected some pathways involved in cell differentiation, proliferation, and female gonadal development that may cause differences in egg production. Examples of these pathways were the PI3K-Akt signaling pathway (*IGF2*, *COMP*, and *FGFR4*), animal organ morphogenesis (*IGF2* and *CDX4*), and female gonad development (*TGFB2*).

**Conclusion:**

On analyzing the genetic background of six local goose breeds by using re-sequencing data, we found that the kinship was consistent with their geographic location. 107 egg-laying trait-associated candidate genes were mined through six selection signal analysis. Our study provides a critical reference for analyzing the molecular mechanism underlying differences in reproductive traits and molecular breeding of geese.

**Supplementary Information:**

The online version contains supplementary material available at 10.1186/s12864-023-09852-x.

## Background

Egg-laying performance is the foundation of the poultry breeding industry. The egg-laying ability directly determines poultry production and the speed and scale of poultry industrialization. Compared with other poultry industries, industrialization of the goose industry has severely slowed down because of low production of egg-laying geese. Therefore, improving egg production is the important goal of goose breeding, and genetics is a key factor affecting goose egg production.

Significant differences exist in egg-laying production of goose germplasm. For example, the annual egg production of high-yield goose germplasm can exceed 100, whereas that of low-yield goose germplasm is only approximately 30. As egg production is influenced by genetics, nutrition and management factors, genetics plays a fundamental role in this process. The key causative factor for this difference is that different goose breeds have been artificially selected for a long time, which has changed the genome region. The imprint on the genome affected by selection is called the selection signal [1, 2]. After several generations of rapid fixation, beneficial mutation sites tend to reduce the genetic variation region upstream and downstream of these sites. Selective signal detection is essential for understanding the origin of livestock breeds and the genetic processes influencing phenotypic differentiation. In addition, detecting selection signals is of great significance for characterizing genetic resources of livestock and identifying genetic variants that lead to economically crucial traits [3].

The development and application of next-generation sequencing (NGS) technology and high-density SNP chips, as well as advanced statistical methods and bio-informatics tools have substantially improved the ability to detect genomic selected regions in livestock and poultry breeds. Selective signal detection is currently among the main research concerns of animal genetics and breeding experts. Numerous studies have unearthed genes and beneficial mutations that exert selection advantages in specific livestock and poultry populations. Li studied the whole genome of nine Chinese chicken breeds at altitudes between 400 and 3000 m. They found that artificial and natural selection have played a gigantic role in the evolution of domestic chickens, and the selected regions of Tibetan chickens carried genes adapted to high altitudes [4]. Zhang performed genome sequencing on two wild and seven domestic duck populations obtained from different parts of China. The domestic ducks had undergone strong artificial selection, especially in plumage, brain and nervous system development, and energy metabolism [5]. On performing selective signal detection analysis on high egg-laying goose, Liu found that the selected candidate genes related to egg production performance [6]. However, because the reference genome adopted by the research institute was not at the chromosome level, locating the candidate gene is impossible. A study conducted the selection signal analysis of Juxian high- and low-yield geese populations [7]. Although some egg production-affecting candidate genes were identified, the common candidate genes among high-yield egg germplasms could not be detected.

In this study, we sequenced the genomes of 55 Chinese indigenous geese from six breeds. ZE goose, originating from Jilin Province, China, is one of the breeds with the highest egg production in China. HY goose, originating from Liaoning Province, China, is a high egg-producing breed. SC goose, originating from Sichuan Province, China, is also a high egg-producing breed. The other three low egg-producing goose breeds, ZD, LH, and ST, primarily selected for meat breeds and originate from Zhejiang Province, Jiangxi Province, and Guangdong Province in China, respectively. These breeds originate from various genetic background and geographic origins. According to the egg production level [8], the geese were divided into the high (more than 60 eggs per year) and low egg-laying (approximately 30 eggs per year) groups. We then performed the inter-population selection signal analysis to explore the selected genome regions in the high egg-laying germplasm population, so as to find the key candidate genes affecting egg-laying traits.

## Results

### Genome resequencing and genetic variation

We combined the original data from 19 blood samples (HY and LH) with 36 database resequencing data (ZE, ZD, SC, and ST) to form the 55 samples (Table 1) in the study. Based on the sequencing results, the average sequencing depth reached 11.75X, with the min and max being 8.07X and 14.94X, respectively (Supplement Table S1). The alignment rate with the reference goose genome was a mean of 98.29%. A total of 5,599,640 SNPs were annotated after quality control and annotation of mutation sites (Table 2). The quality and quantity of SNPs can allow further analysis.

**Table 1:** Information of the goose populations used in this study

Breed	Abbreviation	Sample Size	Egg-laying Production
Huoyan	HY	9	High
Zie	ZE	9	High
Sichuan	SC	10	High
Zhedong	ZD	8	Low
Lianhua	LH	10	Low
Shitou	ST	9	Low

**Table 2:** Characteristics and numbers of identified SNPs for individuals of six breeds

Category		Number of SNPs
Upstream		49,882
Exonic	Stop gain	310
	Stop lose	26
	Synonymous	37,850
	Non-synoymous	16,102
Splicing		182
Downstream		45,191
Upstream/downstream		2662
Intergenic		2,119,882
Transformation		2,373,051
Transversion		954,502
Total		5,599,640

### Population genetics structure and relationships

**Fig. 1:**
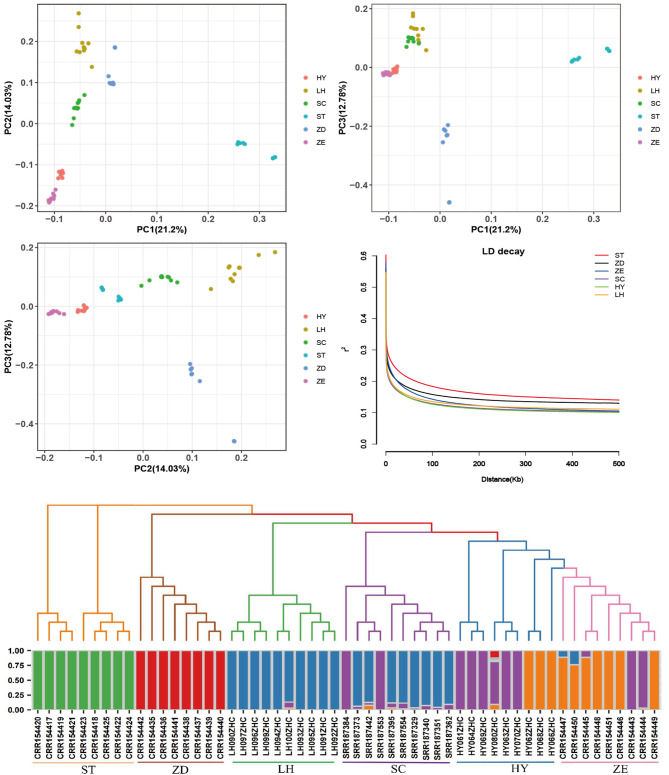
Population genetics structure. (**A**–**C**) Principal component analysis of identified SNPs. (**D**) Linkage disequilibrium (LD) decay. (**E**) Genome-wide admixture analysis

To examine genetic relationships among those six goose breeds, the PCA (Fig. 1A–C), NJ phylogeny and admixture (K = 4) analyses were performed using the whole-genome sequencing data (Fig. 1E). PC1, PC2, and PC3 in the PCA explained that the proportions of total variance were 21.2%, 14.04%, and 12.78%, respectively (Fig. 1A–C). PC1 could clearly separate ST from other breeds. From the perspective of PC2, HY and ZE were closer. To resolve the phylogenetic relationships of those breeds, a NJ tree was constructed among the 55 individuals. When ST was used as a reference population for revealing evolutionary relationships in other breeds, ST was found to be closer to ZD and farthest from ZE, which coincided with the breed’s geographical location. The population structure analysis was performed for detecting the possible ancestry proportions among the breeds. The clustering result for K = 4 indicated the presence of a clear division between the breeds with high egg-laying production (ZE, HY, and SC) and low egg-laying production (ST, ZD, and LH). This is also consistent with the results of the PCA and NJ tree.

To further investigate the degree of selection of different goose breeds, we calculated LD values for each breed (Fig. 1D). According to the LD analysis, ZE had the second longest LD decay distance (half r^2^ value = 2000 bp) of all breeds (Supplement Table S2). These results indicated that ZE was artificially bred at a higher selection intensity than the other four breeds. According to the half r^2^ value of the six breeds, we considered 10 kb as the SNP site-linked distance for the signal detection window.

### Genome-wide selective signature detection

**Fig. 2:**
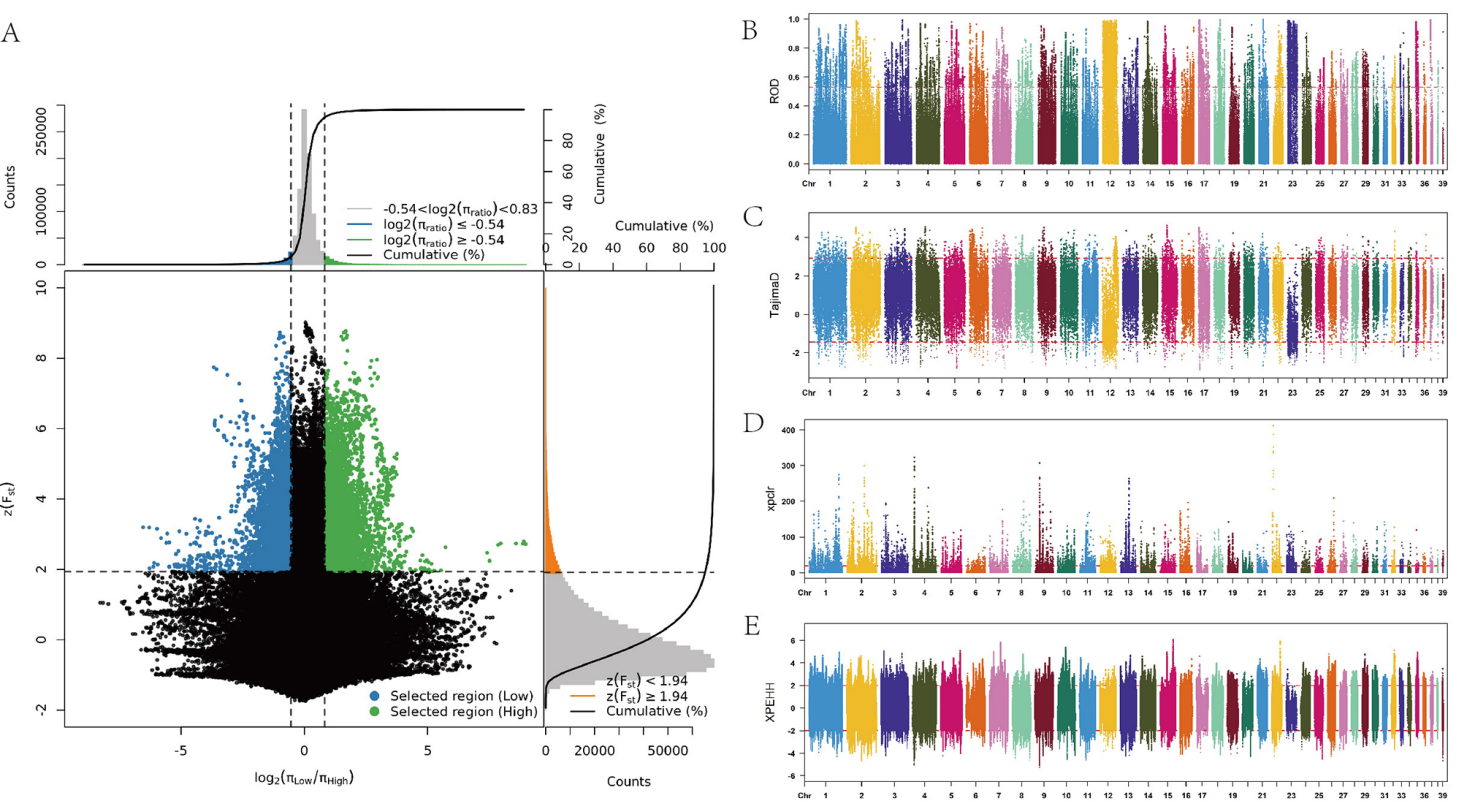
Multiple selection sweep analysis in the high egg production group. (**A**) Fst andπratio selective elimination analyses. (**B**) The ROD plot of selective sweeps in the high group (threshold = 0.53). (**C**) Tajima’s D plot of selective sweeps in the high group (threshold = 2.94 and − 1.45). (**D**) The xpclr plot of selective sweeps in the high group (threshold = 18.89). (**E**) The XPEHH plot of selective sweeps in the high group (threshold = 2 and − 2)

To better mine the functional areas closely related to egg-laying traits, we assigned three breeds (HY, ZE, SC, total 28 samples) to the high egg-laying group, and other three breeds (ZD, LH, ST, total 27 samples) to the low egg-laying group. Considering that a single method may generate false-positive selection signals, the results of F_ST_ and π_ratio_ analysis methods were combined to screen out the sites that appeared in the top 5% window of the Z(F_ST_) value (≥ 1.94) sliding window analysis and the top 5% window of the log2(π_ratio_) value ( ≥ − 0.54) analysis as the high-group selected candidate target sites (Fig. 2A). More windows of selection (1411 and 1119) were present on Chr1 and Chr17, respectively. Among them, the region of Chr1:63306001–63,316,000 exhibited the highest F_ST_ value of 8.77. We next identified the high and low group-selected genomic regions by comparing the ROD (reduction of diversity) using a 10-kb window with a 1-kb step size (Fig. 2B). The top 5% of windows or regions with the highest ROD value (threshold ≥ 0.53) were defined as the high group sweep. More windows of selection (6287 and 4356) were present on Chr12 and Chr23, respectively. The region of Chr12:18159001–18,169,000 exhibited the highest ROD value of 0.99. Tajima’s D, which is based on allele frequencies, was used to detect the high group-selected regions, the Tajima’s D value (threshold ≥ 2.94 and ≤ -1.45)as the selected region (Fig. 2C). Chr12 exhibited the largest distribution of selected regions (816 SNPs). Using the cross-population composite likelihood ratio test, we selected the top 5% window as the high group-selected region, which had a xpclr value of ≥ 18.89 (Fig. 2D). The region with the highest values (32.02) appeared on Chr15:10575001:10585000. Chr2 exhibited the largest distribution of selected regions (3221 SNPs). We further performed selective signal detection on the high group by using the inter-population haplotype-based XP-EHH method (Fig. 2E). The xpehh value of sites on the high group-selected region was between ≥ 2 and ≤ − 2, and the largest number of sites was observed on Chr2, accounting for 15.5% of all chromosomes. Overall, the six selective signal detection results revealed that the most selected regions were located on Chr2 and Chr12. Furthermore, the significant regions and loci identified by each method, as well as the loci shared among all six methods, methods were detailed list in supplementary Table S3.

### Candidate gene enrichment analysis

**Fig. 3:**
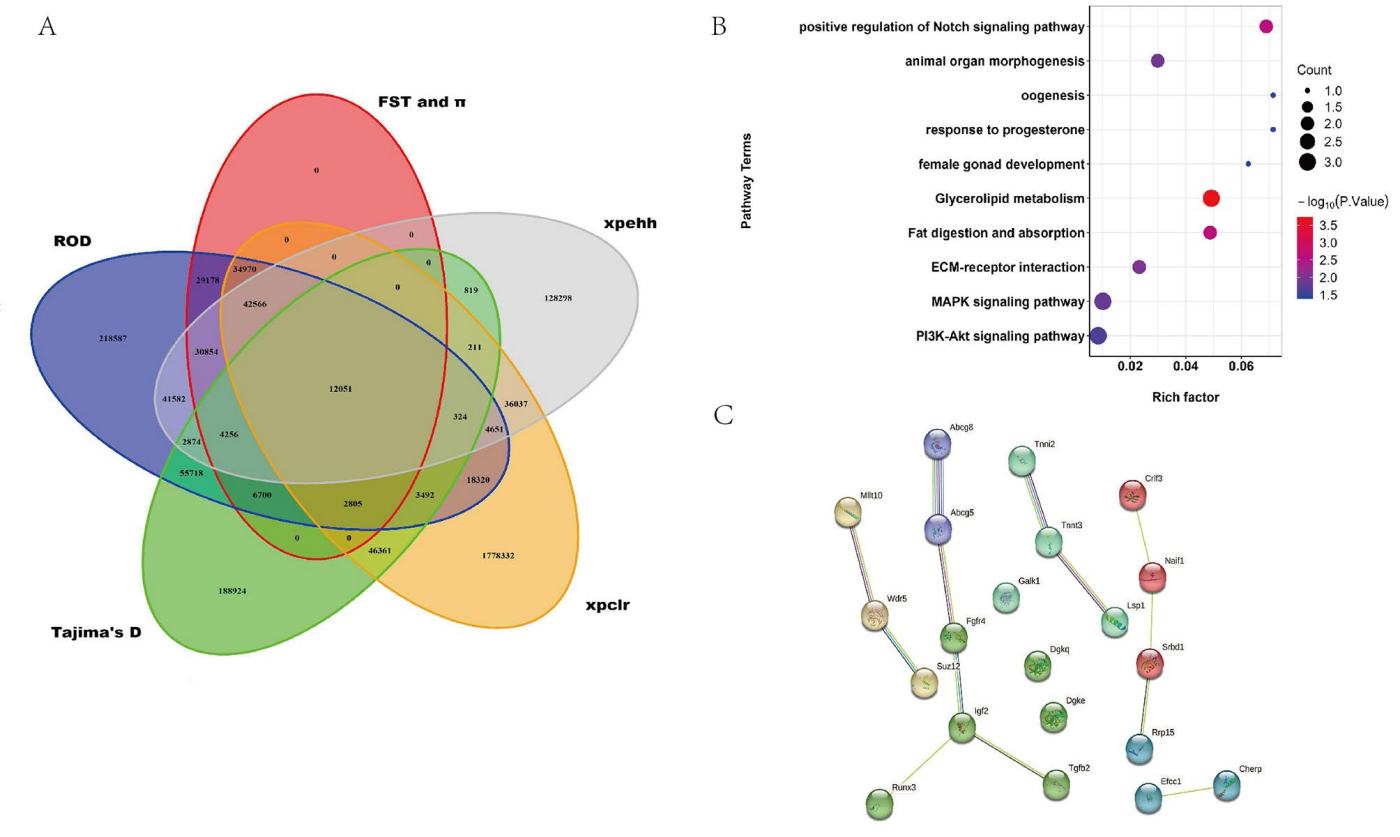
Candidate gene function enrichment and protein interaction analyses. (**A**) Venn diagram showing the SNPs overlap among Fst andπ, ROD, Tajima’s D, xpclr, and xpehh. (**B**) Bubble plot of the candidate gene function enrichment pathway. (**C**) The protein interaction diagram of the candidate gene

We performed Venn analysis of SNP sites in selected regions found in the high egg-laying group among the six methods. A total of 12,051 SNP sites were selected in all six methods (Fig. 3A). Then, annotation of these common SNP sites resulted in 107 candidate genes. To better evaluate the candidate gene functions, GO and KEGG enrichment analyses were performed on these genes. The enrichment results (Table 3) revealed some pathways (Fig. 3B) involved in cell differentiation, proliferation, and female gonadal development, such as the PI3K-Akt signaling pathway (*IGF2, COMP*, and *FGFR4*), animal organ morphogenesis (*IGF2 and CDX4*), and female gonad development (*TGFB2*).

**Table 3:** Results of enrichment analysis for the candidate genes of egg-laying production

Database	Term	ID	*P* Value	Enrich Genes (Position)
KEGG	Glycerolipid metabolism	hsa00561	0.0002	*DGKQ* (Chr23), *GK* (Chr16), *DGKE* (Chr28)
KEGG	Fat digestion and absorption	hsa04975	0.0025	*ABCG8* (Chr10), *ABCG5* (Chr10)
KEGG	ECM–receptor interaction	hsa04512	0.0100	*COMP* (Chr35), *GPIX* (Chr18)
KEGG	MAPK signaling pathway	hsa04010	0.0145	*IGF2* (Chr2), *FGFR4* (Chr24), *TGFB2* (Chr10)
KEGG	PI3K-Akt signaling pathway	hsa04151	0.0232	*IGF2* (Chr2), *COMP* (Chr35), *FGFR4* (Chr24)
GO	Positive regulation of the Notch signaling pathway	GO:0045747	0.0026	*TGFB2* (Chr10), *ASCL1* (Chr3)
GO	Animal organ morphogenesis	GO:0009887	0.0122	*IGF2* (Chr2), *CDX4* (Chr22)
GO	Oogenesis	GO:0048477	0.0358	*NAIF1* (Chr29)
GO	Response to progesterone	GO:0032570	0.0358	*TGFB2* (Chr10)
GO	Female gonad development	GO:0008585	0.0405	*TGFB2* (Chr10)

We used the STRING tool for the protein interaction network analysis of proteins encoded by the candidate genes (Fig. 3C) and obtained five subnetworks. The largest subnetwork included six nodes and five edges, composed of *ABCG8, ABCG5, FGFR4, IGF2, TGFB2*, and *RUNX3* genes, and the pivot gene was *IGF2*.

## Discussion

The characterization of the population structure and genomic comparisons between closely related species are essential for genetic assessment, as well as for the utilization and conservation of goose breed genetic resources. We here performed whole-genome sequencing of 55 Chinese indigenous geese from six breeds that belonged to the low egg-laying to high egg-laying production groups. We explored the population genetic structure of six breeds by using PCA, NJ tree, and STRUCTURE. The genetic background analysis exhibited that the six goose breeds had a relatively separate genetic background. Overall, the partitioning of genetic diversities of the breeds was consistent with their geographic distributions. Although the genetic backgrounds of the six goose breeds are relatively independent, there is also a certain common ancestor between the breeds. For example, ZE and HY were genetically close and their blood composition was similar. SC with a high egg-producing trait shared part of the ancestral lineage with ZE and HY (Fig. 1E). ZD, LH, and ST had less exchange of blood. This may be related to their obvious physical features and mountainous separation. In general, the background analysis results were consistent with the formation history of each breed.

Regarding the mining of egg-laying trait genes of goose, most predecessors have conducted the population association analysis based on polymorphisms of a single candidate gene. Only a few studies have conducted population association analysis based on polymorphisms at the genome-wide level. Liu used various selection signaling methods for analyzing the egg-producing traits of geese [6]; However, limited by the assembly level of the reference genome version, locating the chromosome on which the candidate gene is located is impossible. In this study, the latest version of the chromosome-level reference genome was used for detecting genome-wide variation. Because the latest version can accurately identify the location of the variation interval, which is more advantageous in mining new variants. Humans improve the egg-laying ability of high-laying geese through long-term artificial selection, and determining which genes are selected under long-term selection conditions is the key to studying the genes that affect egg-producing traits.

Since the probability of finding a false-positive interval is high with the single-choice signal method, various selection signal analysis methods (Fst, π, ROD, Tajima’s D, xpclr, and xpehh) were used for detecting population selection signals in the goose germplasm with significant differences in egg production performance. These methods also allowed exploration of candidate genes that affect egg production performance. In the aforementioned signal analysis methods, we established the selection defining criteria generally considered in the research field as the threshold of the selected region. For example, top 5% values included Fst, π, ROD, and xpclr; top 2.5% and bot 2.5% values included Tajima’s D; and the exceeding threshold line (value = 2 and − 2) of xpehh. To identify the common SNPs in the selected regions, we performed Venn analysis of 12,051 SNP sites selected in the six methods. These candidate SNPs were annotated to a total of 107 genes, and an average of more than 100 sites were annotated to a gene. To better comprehend the function of candidate genes, we performed the functional enrichment analysis and protein interaction network analysis.

According to our results, the candidate genes were significantly enriched in the pathways related to female organ development and cell proliferation and differentiation, such as the mitogen-activated protein kinase (MAPK) signaling pathway (hsa04010), PI3K-Akt signaling pathway (hsa04151), response to progesterone (GO:0032570), animal organ morphogenesis (GO:0009887), oogenesis (GO:0048477), and female gonad development (GO:0008585). The candidate genes contained in the aforementioned pathways were mainly *IGF2*, *FGFR4*, *TGFB2*, and *NAIF1*.

The MAPK signaling pathway is a well-conserved intracellular signal transduction pathway in eukaryotes, playing a vital role in cell proliferation, differentiation, apoptosis, and metabolism [9]. It is also crucial for animal reproduction. Inhibition of MAPKK-MAPK members and ERK pathway scaffold proteins transcription effectively halts insect ovarian development, leading to immature ovaries, reduced egg production, and complete inhibition of fecundity, highlighting the critical role of the MAPK signaling pathway in female insect reproduction [10].

The PI3K-Akt signaling pathway regulates various cellular processes, including metabolism, proliferation, cell survival, growth, and angiogenesis, in response to extracellular signals. In poultry, follicular atresia increases with age, leading to reduced fecundity. Thus, preventing follicular atresia is essential for maintaining high egg production in poultry. Inhibiting the PI3K/AKT pathway in the insulin signaling pathway can accelerate granulosa cell (GC) apoptosis and result in premature ovarian failure. In the poultry industry, an increase in the number of atretic follicles (AFs) directly reduces egg production. By contrast, reducing the number of AFs increases the chance of development of follicles into graded follicles, thereby increasing egg production [11]. Therefore, the PI3K-Akt signaling pathway significantly influences poultry egg-laying traits. Studies on laying hen fecundity have identified differentially expressed genes (DEGs) associated with high laying rates, particularly those involved in the regulation of the PI3K-Akt signaling pathway in the hypothalamic-pituitary-gonadal (HPG) axis [12]. Similarly, research in Muscovy ducks has demonstrated the importance of the PI3K-Akt signaling pathway and ovarian steroid synthesis in follicle development and fecundity [13]. Additionally, the activated PI3K/Akt/mTOR signaling pathway plays a crucial role in the proliferation and anti-apoptosis of granulosa cells in geese [14].

The progesterone receptor (PR) is a nuclear receptor for progesterone that plays a crucial role in various aspects of female development and reproduction. PR expression significantly decreases during the nesting stage [15]. Specific hormone response and pituitary transcriptional regulation in the spawning and hatching stages of Muscovy duck [16]. Ovarian differentiation and maturation are integral to the complex process of gonadal development, which directly impacts fertility and reproductive success later in life. This study focused on gonadal development, suggesting a potential link between differences in egg-laying performance among germplasms and embryonic stage gonadal development.

Insulin-like growth factor 2 (*IGF2*) has a vital role in follicular development. In mammals, *IGF2* is highly expressed in dominant follicles, thereby supporting key functions of follicular development [17]. IGF2 can stimulate the proliferation of granulosa cells and synthesis of related hormones, and regulate follicular development through FSH [18], thereby affecting the fecundity of sows and cattle [19, 20]. Furthermore, *IGF2* expression in the rat ovary directly affects the development of dominant follicles [21]. The luteinizing hormone and FSH also regulate the ovarian function of birds. *IGF2* is widely expressed in different chicken tissues, but the highest expression is observed in ovaries. Follicular *IGF2* expression was significantly higher in high-yielding chickens than in low-yielding chickens. A certain relationship was observed between ovarian *IGF2* expression and egg production [22]. Similarly, IGF2 is implicated in the follicular development of Muscovy ducks and regulates oviposition. The linkage loci Amur1864G and Cmer1704G of IGF2 are significantly correlated with E59W, indicating a positive association between IGF2 and egg-laying traits [23]. These in vivo and in vitro studies highlight the critical role of IGF2 in ovarian follicular development [24] and its relevance to poultry egg-laying traits.

FGFR4, a protein-coding gene, plays a regulatory role in various pathways, including cell proliferation, differentiation, migration, and vitamin D metabolism. In the kidney, FGFR4 gene expression is significantly higher during the peak laying period compared to the early and late stages [25]. Within bovine granulosa cells, FGFR4 mRNA levels increase in medium-sized E2-inactivated follicles during follicle selection and innervation, suggesting its involvement in preventing differentiation of antral follicles of this size [26]. Interestingly, several fibroblast growth factors that preferentially bind to FGFR1c, FGFR2c, FGFR3c, and FGFR4 seem to serve as key regulators of large follicular differentiation and atresia. For example, *FGF2* (preferentially binds to FGFR1c and FGFR3c), *FGF9* (preferentially binds to FGFR3c and to FGFR2c), and *FGF17* and *FGF18* (preferentially binds to FGFR3c and then to FGFR4) inhibit steroid synthase activity and the production of GC-mediated stimulation of FSH-stimulated E2. The *FGFR4* protein is expressed in all follicular types of oocytes [27].

Transforming growth factor β 2 (*Tgfb2*)is a protein-coding gene. *Tgfb2* is mainly expressed in mammalian ovarian cells. The *Tgfb2* gene and protein are expressed in granulosa cells; membrane cells of bovine [28]; human, rat, and mouse ovaries; and mouse oocytes. *Tgfb2* was highly expressed in the ovary on the 4th day after birth, when the ovary mainly contained primordial follicles, primary follicles, and progenitor cells [29]. The aforementioned studies have shown that *Tgfb2* is crucial for follicular development and can be used as a key candidate gene for studying follicular development.

## Conclusions

This study provides a comprehensive overview of genomic variations in goose by using WGS data. The kinship relationship between high and low egg-laying goose germplasms was clarified. The relationship was consistent with the geographical location of distribution. In addition, using six selection signal analysis methods, we identified 107 candidate genes related to the egg-producing traits. These candidate genes offer an important research basis for further elucidating the formation mechanism of goose egg production.

## Methods

### Samples and re-sequence data

The blood samples of 9 HY and 10 LH geese were collected from the Taizhou waterfowl breeding farm, and 5 mL sublingual vein blood was drawn into blood collection tubes containing the EDTA-K2 anticoagulant. The birds were released after their blood was sampled. By employing the CWE9600 Magbead Blood DNA Kit, DNA was extracted using the magnetic bead method. Paired-end libraries with an insert size of 350–500 bp were constructed for each bird. After the library inspection was qualified, pooling was performed based on the effective library concentration and the demand for the target data volume. The sequencing method of PE150 was selected using the DNBSEQ-T7 sequencer for machine sequencing.

To profoundly explore the common genes of high and low egg production traits, we collected additional 36 samples by referring to the raw sequence database uploaded by previous studies [6, 30]. These 36 samples included ZE (*n* = 9), SC (*n* = 10), ZD (*n* = 8), and ST (*n* = 9). In total, 55 bird samples were used in this study. All samples were divided into the high egg-laying production (HY, ZE, and SC) and low egg-laying production (ZD, LH, and ST) groups.

### SNP calling and annotation

To ensure the quality of the sequencing data, fastp was used to perform a series of quality control checks for raw reads following standard procedures [31]. The raw data were filtered in accordance with the following conditions:

1. Reads containing the linker sequence were filtered;
2. The N content in single-ended access reads exceeding 10% of the read length was set as the standard for deleting paired reads;
3. When the number of low-quality (≤ 5) bases contained in the single-ended sequencing read exceeded 50% of the length of the read length, the paired reads were removed.

After data were filtered, the index was built using the goose chromosome-level reference genome version [32]. Then, clean reads were compared to the reference genome by using BWA 0.7.17 software [33], sorted, and indexed using samtools1.7. The bam file was deduplicated using the module provided with GATK 4.1.8.0 software [34]. Then, the sequencing depth, genome coverage, and other information of each sample were calculated based on the bam file.

By using GATK 4.1.8.0 software to call SNPs, SNP mutations were detected based on the comparison results of clean reads in the reference genome. Then, the SNP standard was filtered using the variant filtration module. The nucleotide variants were filtered based on the quality requirement with the read depth (dp > 2), missing rate (Miss < 0.1), and MAF (> 0.05) using the SAMtools. Finally, SNP variant sites were annotated using annovar software.

### Population genetics analysis

In this study, principle component analysis (PCA) was performed based on all SNPs. The first three principal components of the population (parameter --pca 3) were calculated using Plink software (version: 1.9) [35]. The distribution plots of PC1–PC2, PC1–PC3, and PC2–PC3 samples were mapped using the “ggplot2” package in R software.

The identity-by-state (IBS) genetic distance matrix (parameter -- distance 1-ibs square) was constructed using Plink software (version: 1.9). Using the neighbor-joining (NJ) method, the evolutionary tree was constructed with MEGACC software. Finally, the Interactive Tree Of Life online tool was used to visualize the evolutionary tree results to present the evolutionary relationship between individuals of the six goose breeds.

The computationally efficient admixture software was used to analyze the population structure of the six goose breeds [36]. The parameters were set by software default settings, and the number of subgroups was K = 2– 6 for simulation calculation. The “pophelper” package in R software was used to map the structure of subgroups to study the stratification of all populations [37].

The linkage disequilibrium (LD) decay with physical distance between SNPs was calculated and visualized using PopLDdecay software [38] with the parameter (--MaxDist 500).

### Detection of selective signatures

Genome scans for selection in the high egg-laying population were performed using five methods and 4 strategies. First, based on population differentiation, VCFtools [39] were used to calculate the fixation index (Fst) between the high and low group breeds. Based on the LD results, the window was finally set to 10 kb and the step size to 1 kb. Second, based on genomic heterozygosity π_ratio_ and ROD, π_low_/π_high_ was calculated using a 10-kb window with a 1-kb step size, and the top 5% of windows, we identified genomic regions selected by the high and low groups by comparing the ROD using a 10-kb window with a 1-kb step size. The top 5% of windows or regions with the highest ROD value were defined as the high group sweep. Third, based on allele frequency profiles, Tajima’s D value was calculated with VariScan (version 2.0.3) by using a 100-kb window and a 10-kb step size. The cross-population composite likelihood ratio (xpclr) was calculated for sites in the 10-kb window with a 1-kb step size of each chromosome by using xpclr software [40]. Fourth, based on LD, the inter-population XP-EHH analysis was performed using Shapit software to construct genome haplotype information, and the rehh package (version 3.1.2) in R software was to perform [41]. A selective signal detection method based on population differentiation (F_ST_) and genomic heterozygosity (π_ratio_) was used for combinatorial analysis, so that F_ST_ and π_ratio_ could be mutually validated to avoid false positives and screen overlapping sites detected by combining the two methods. To facilitate the overlapping sites detected, the obtained F_ST_ and π_ratio_ values were converted to ZF_ST_ and Zπ_ratio_ values through standard normal conversion (Z-transformed). The ZF_ST_ and Zπ_ratio_ values were then sorted, and the overlap region with the selection signal was presented as the selected candidate region in the top5% regions of both methods.

### Candidate gene functional annotation

For a better understanding of the gene functions and signaling pathways of the identified candidate genes, online GO and KEGG pathway enrichment analyses were performed using KOBAS 3.0. Candidate genes in key pathways were analyzed using the STRING database (https://string-db.org/) for protein interaction network analysis.

### Electronic supplementary material

Below is the link to the electronic supplementary material.

**Supplementary Material 1: Table S1.** The statistical table of sequencing results for each sample.

**Supplementary Material 2: Table S2.** The statistics of LD decay from six breeds.

**Supplementary Material 3: Table S3.** The detailed list of selected region mined by six selective signal analysis methods.

## Data Availability

Sequences are available from the CNCB-NGDC with the Bioproject accession numbers PRJCA018930.

## Abbreviations

WGS: Whole-genome sequencing
Chr: Chromosome
ZE: Zi goose
HY: Huoyan goose
SC: Sichuan goose
ZD: Zhedong goose
LH: Lianhua goose
ST: Shitou goose
F_ST_: Fixation index
ROD: Reduction of diversity
XP-EHH: Cross-population extended haplotype homozygosity
PCA: Principle component analysis
GO: Gene ontology
KEGG: Kyoto encyclopedia of genes and genomes
